# Radiotherapy Suppresses Bone Cancer Pain through Inhibiting Activation of cAMP Signaling in Rat Dorsal Root Ganglion and Spinal Cord

**DOI:** 10.1155/2016/5093095

**Published:** 2016-02-18

**Authors:** Guiqin Zhu, Yanbin Dong, Xueming He, Ping Zhao, Aixing Yang, Rubing Zhou, Jianhua Ma, Zhong Xie, Xue-Jun Song

**Affiliations:** ^1^Center for Clinical Research and Translational Medicine, Lianyungang Oriental Hospital, Lianyungang, Jiangsu 222042, China; ^2^Department of Anesthesiology, Lianyungang Oriental Hospital, Lianyungang, Jiangsu 222042, China; ^3^Division of Anesthesiology & Pain Medicine, Key Laboratory of Carcinogenesis and Translational Research, Ministry of Education of China, Beijing Cancer Hospital, Peking University, Beijing 100142, China; ^4^Department of Anesthesiology, Beijing Cancer Hospital, Peking University, Beijing 100142, China

## Abstract

Radiotherapy is one of the major clinical approaches for treatment of bone cancer pain. Activation of cAMP-PKA signaling pathway plays important roles in bone cancer pain. Here, we examined the effects of radiotherapy on bone cancer pain and accompanying abnormal activation of cAMP-PKA signaling. Female Sprague-Dawley rats were used and received tumor cell implantation (TCI) in rat tibia (TCI cancer pain model). Some of the rats that previously received TCI treatment were treated with X-ray radiation (radiotherapy). Thermal hyperalgesia and mechanical allodynia were measured and used for evaluating level of pain caused by TCI treatment. PKA mRNA expression in dorsal root ganglion (DRG) was detected by RT-PCR. Concentrations of cAMP, IL-1*β*, and TNF-*α* as well as PKA activity in DRG and the spinal cord were measured by ELISA. The results showed that radiotherapy significantly suppressed TCI-induced thermal hyperalgesia and mechanical allodynia. The level of PKA mRNA in DRG, cAMP concentration and PKA activity in DRG and in the spinal cord, and concentrations of IL-1*β* and TNF-*α* in the spinal cord were significantly reduced by radiotherapy. In addition, radiotherapy also reduced TCI-induced bone loss. These findings suggest that radiotherapy may suppress bone cancer pain through inhibition of activation of cAMP-PKA signaling pathway in DRG and the spinal cord.

## 1. Introduction 

Pain is one of the most prevalent symptoms in patients with primary bone sarcomas and with the distant metastases of nonbone primary tumors [1, 2]. Studies have indicated that bone cancer pain has complex and unique mechanisms that may involve both inflammatory and neuropathic pain [3]. Clinically, most patients with bone cancer have already passed the optimal time for radical surgery and multidisciplinary therapies. However, with radiotherapy, majority of these patients experience pain relief, control of tumor growth, and prolonged survival. Radiotherapy is not only an effective method in the clinical treatment of bone cancer, but also an important approach for treatment of the severe pain associated with bone cancer. However, mechanisms underlying radiotherapy for bone cancer pain have not been well investigated and remain elusive.

Cyclic adenosine monophosphate-protein kinase A (cAMP-PKA) signaling pathway plays important roles in a number of cellular processes, including immune function [4], growth [5], differentiation [6], and metabolism [7], and is essential to the plasticity in neural synapses in CNS [8]. Activation of cAMP-PKA pathway has been reported to enhance presynaptic neurotransmitter synthesis and vesicular transportation probably through phosphorylation of key transcriptional factors (i.e., cAMP response element-binding protein) and synaptic vesicle proteins [9–11]. In addition, recent studies found that cAMP-PKA signaling pathway is involved in both inflammatory pain [12–14] and neuropathic pain [15–17]. The peripheral hyperalgesic actions of inflammatory mediators are mediated by the cAMP-PKA signaling pathway [18]. We have recently demonstrated that the cAMP-PKA pathway is crucial for the maintenance of dorsal root ganglia (DRG) neuronal hyperexcitability and behaviorally expressed hyperalgesia, in an* in vivo* neuropathic pain animal model of chronic compression of the DRG (CCD model), as well as in an* in vitro* model of acute DRG dissociation [15, 16, 18]. Recently, we have further found that activation of the cAMP-PKA signaling pathway plays an important role in both induction and maintenance of bone cancer pain in rats [19]. However, it remains unknown whether and then how cAMP-PKA signaling would contribute toward radiotherapy treatment for bone cancer pain. This study provides evidence supporting an idea that radiotherapy may suppress bone cancer pain through inhibition of abnormal activation of cAMP-PKA signaling pathway in DRG and the spinal cord.

## 2. Materials and Methods

### 2.1. Animals and Drugs

Female adult Sprague-Dawley rats (160–180 g at the start of the experiment) were housed in a controlled lighting environment with free access to food and water. All experiments were approved by the Institutional Animal Care and Use Committees in Oriental Hospital and conducted in accordance with the Declaration of the National Institutes of Health* Guide for the Care and Use of Laboratory Animals* (publication number 85-23, revised 1985). Surgery was performed under anesthesia with intraperitoneal injection of sodium pentobarbital (50 mg/kg, i.p.).

### 2.2. Animal Model of Bone Cancer Pain

The protocols of the bone cancer pain model were similar to that described previously [19–21]. In brief, following induction of general anesthesia with intraperitoneal injection of sodium pentobarbital, rats were placed abdominal side up. After disinfecting with 75% v/v ethanol, a one-centimeter rostrocaudal incision was made in the skin directly above the top half of the tibia. Tumor cells (1 × 10^5^ cells/*μ*L, 5 *μ*L), extracted from the ascetic fluid of female rats that received Walker 256 mammary gland carcinoma cells, were injected (tumor cell implantation, TCI) into the intramedullary space of the right tibia to induce bone cancer. Injection site was closed with bone wax while the syringe was removed. The incision was then dusted with penicillin powder and closed. Rats in the sham group were injected with the same number of boiled tumor cells.

### 2.3. Radiotherapy

A single dose of radiation was given 9 days after TCI. Rats were immobilized in an acryl jig, and a dose of 6 Gy was delivered to the right tibia area using a collimator system with 6 MV X-rays [22]. Sham radiotherapy applied in TCI rats was using the same protocol, except that they were not given the real X-ray radiation.

### 2.4. Radiographic Observation

On the 17th day after TCI treatment, radiographic images were taken (exposure setting: 12 ms, 31 KVp) using a Philips Digital Radiographer System (Digital Diagnost VM; Philips Medical Systems DMC GmbH, Hamburg, Germany). Bone destruction was evaluated on a scale of 0–5 [20, 23]: 0 = normal bone structure without any sign of deterioration; 1 = small radiolucent lesions in the proximal epiphysis (<3); 2 = increased number of radiolucent lesions (>3) indicating loss of medullary bone; 3 = loss of medullary bone, plus erosion of the cortical bone; 4 = full-thickness unicortical bone loss; and 5 = full-thickness bicortical bone loss and displaced fracture. The experimenter was blinded to the treatment of the samples.

### 2.5. Behavioral Test

Thermal hyperalgesia was indicated by a significantly shortened latency of foot withdrawal in response to heat stimulation. To determine thermal hyperalgesia, a radiant heat source was focused and delivered on a portion of the hind paw; the thermal stimuli shut off automatically when hind paw moved (or after 20 s to prevent tissue damage). Thermal stimuli were delivered 3 times to each hind paw at 5–8-minute intervals. Mechanical allodynia was indicated by a significant decrease in the threshold of paw withdrawal to mechanical indentation of the plantar surface of each hind paw, with a sharp, cylindrical probe. The probe was applied to 6 designated loci distributed over the plantar surface of the foot. The minimal force that induced paw withdrawal was read off the display [19, 24]. The experimenters who performed these behavioral tests were blinded to the treatment condition of the animals.

### 2.6. mRNA Isolation and RT-PCR

The total RNA isolated with TRIzol Reagent (Invitrogen, USA) was reverse transcribed using M-MLV reverse transcriptase (Takara). Primer sets were synthesized by Integrated DNA Technologies (Sangon Biotech); their sequences are shown in Table 1. The amplification conditions were set as follows: 94°C for 2 min and 30 cycles of 94°C for 30 s and 58°C for 40 s. PCR products were analyzed on agarose gel electrophoresis and were verified by DNA sequencing. The gels were imaged with Tanon 2500 Imaging Systems and analyzed by analysis software ImageJ 1.48u.

### 2.7. Measurement of cAMP, IL-1*β*, and TNF-*α* Levels and PKA Activity

The DRG and the spinal cord at segments of L_4_-L_5_ ipsilateral to TCI were collected on postoperative days 10 and 14 for further neurochemical analysis. Commercial enzyme-linked immunosorbent assay kits were used to determine the concentrations of cAMP, IL-1*β*, and TNF-*α* and activity of PKA, according to the manufacturers' instructions. ELISA kit for cAMP was purchased from Cayman Chemical (Ann Arbor, Michigan, USA); ELISA kits for IL-1*β*, TNF-*α*, and activated PKA were purchased from R&D Systems (Minneapolis, Minnesota, USA).

### 2.8. Statistical Analysis

All statistical analyses were carried out using Statistical Product and Service Solutions, ver. 15.0 (SPSS Inc., Chicago, Illinois, USA). Alterations in cAMP mRNA and concentration, PKA activity, and the levels of IL-1*β* and TNF-*α* were tested using one-way analysis of variance (ANOVA) followed by Bonferroni* post hoc* tests. Two-way repeated-measures ANOVA (days × groups) was used to test the behavioral responses to thermal and mechanical stimuli, followed by Bonferroni* post hoc* tests. The nonparametric statistical method was used for analyzing the bone radiographs. All data were presented as means ± SEM. Statistical results were deemed significant if *P* value was less than 0.05.

## 3. Results

### 3.1. Radiotherapy Attenuated TCI-Induced Thermal Hyperalgesia and Mechanical Allodynia

**Figure 1:**
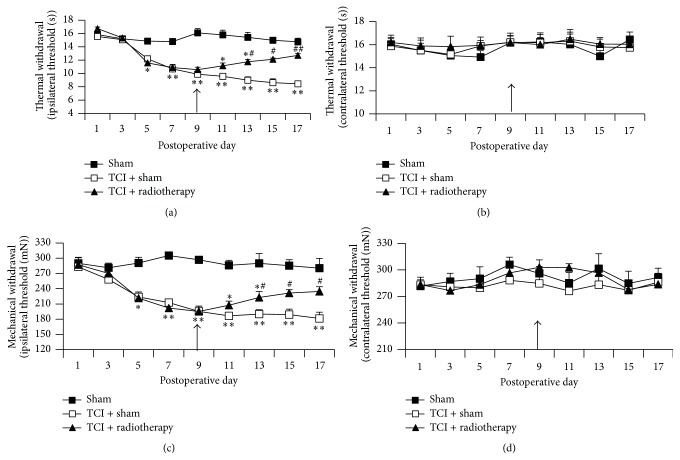
Radiotherapy relieved thermal hyperalgesia and mechanical allodynia in TCI rats. Thermal hyperalgesia of the hind paw ipsilateral (a) and contralateral (b) to TCI. Mechanical allodynia of the hind paw ipsilateral (c) and contralateral (d) to TCI. Arrows indicate administration of X-radiation (6 Gy) on the 9th day after surgery. Eight rats were included in each group. ^*∗*^ *P* < 0.05, ^*∗∗*^ *P* < 0.01 versus sham; ^#^ *P* < 0.05, ^##^ *P* < 0.01 versus TCI + sham radiotherapy.

TCI-treated rats exhibited significant thermal hyperalgesia and mechanical allodynia during postoperative days 5–17. The thresholds of thermal and mechanical withdrawal ipsilateral, but not contralateral, to the TCI treatment decreased approximately by 50% during postoperative days 9–17 compared to sham control. To determine whether radiotherapy treatment affects bone cancer pain, a single dose of X-radiation (6 Gy) was applied on the 9th day after operation. Such radiotherapy produced long-lasting inhibition of TCI-induced thermal hyperalgesia and mechanical allodynia. The inhibition lasted for a week with 1 d delay following the X-radiation application. The peak values of inhibition of the thermal hyperalgesia and mechanical allodynia were approximately 25–50%. The thermal and mechanical withdrawal of the hind paw contralateral to TCI treatment was not altered following the radiotherapy. Data are summarized in Figure 1.

### 3.2. Radiotherapy Alleviated TCI-Induced Bone Destruction

**Figure 2:**
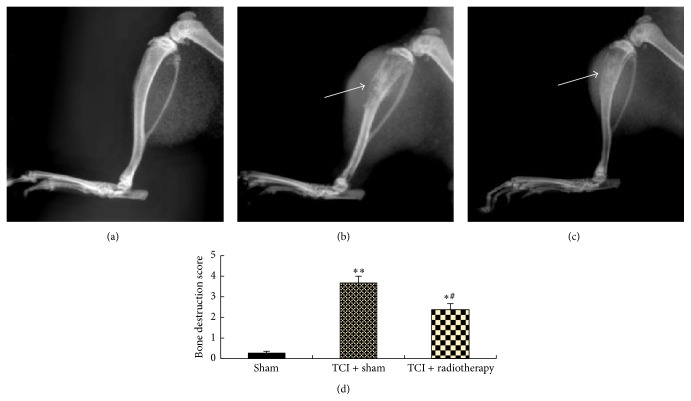
Radiotherapy alleviated TCI-induced tibia bone destruction. Radiographic images were taken on the 17th day after TCI. (a, b, c) Representative bone destruction and tumor growth in groups of sham (a), TCI + sham radiotherapy (b), and TCI + radiotherapy (c). Data are summarized in (d). Eight rats were included in each group. Arrows in (a–c) point to the areas of the bone destruction. ^*∗∗*^ *P* < 0.01 versus sham; ^#^ *P* < 0.05 versus TCI + sham; ^*∗*^ *P* < 0.05 versus sham.

Bone structure integrity was evaluated by radiographic observation on the 17th day after TCI. No radiographic change (score = 0, no bone destruction) was found in the group of sham without radiotherapy. Bone destruction was seen clearly in groups of TCI with and without radiotherapy, respectively. Score of the TCI group with sham therapy was 3.75 ± 0.43 (range: 3–5). Radiotherapy greatly reduced TCI-induced tibia bone destruction. The score in the group of TCI with radiotherapy dropped to 2.38 ± 0.52 (range: 1–3), which was significantly less than the score in TCI group with sham therapy. Radiological changes of the tibia are shown in Figure 2.

### 3.3. Radiotherapy Inhibited TCI-Induced PKA mRNA Expression in DRG

**Figure 3:**
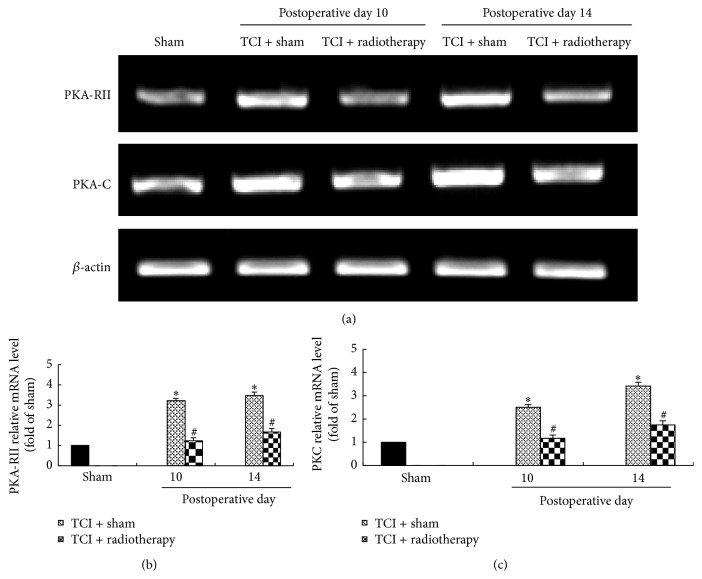
Levels of PKA-RII and PKA-C mRNA in DRG. (a) Representative bands showing levels of PKA-RII and PKA-C mRNA analyzed by RT-PCR. (b and c) Data quantification. Four samples were used for each group with two ganglia in each sample. ^*∗*^ *P* < 0.05 versus sham; ^#^ *P* < 0.05 versus TCI + sham.

Our previous studies have shown that expression of PKA-RII and PKA-C mRNAs was increased after TCI treatment in a time-dependent manner [19]. To test whether radiotherapy has an effect on this TCI-induced PKA mRNA expression, we measured the levels of PKA-RII and PKA-C mRNAs on day 1 and day 5 after radiotherapy treatment (postoperative days 10 and 14, resp.) using RT-PCR. We found that radiotherapy treatment greatly inhibited TCI-induced increase of expression of PKA-RII and PKA-C mRNA. The expression of PKA-RII and PKA-C mRNA in the TCI + radiotherapy group was significantly reduced compared with the TCI + sham group (Figure 3).

### 3.4. Radiotherapy Reduced TCI-Induced Increase of cAMP Level and PKA Activity in DRG and the Spinal Cord

**Figure 4:**
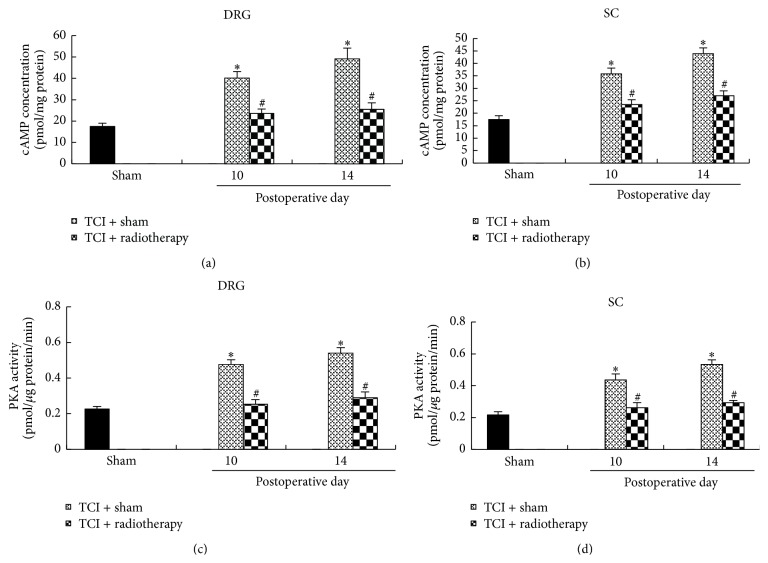
Alterations of cAMP concentration and PKA activity in DRG and the spinal cord after TCI with or without radiotherapy. (a and b) cAMP concentration. (c and d) PKA activity. The tissues were collected on postoperative days 10 and 14, that is, 1 and 5 days after radiotherapy, respectively. Each group included four samples with two ganglia in each sample. ^*∗*^ *P* < 0.05 versus sham; ^#^ *P* < 0.05 versus TCI + sham.

We have previously shown that cAMP concentration and PKG activity in DRG and the spinal cord were significantly increased in a time-dependent manner after TCI treatment [19]. To test the hypothesis that cAMP-PKA signaling pathway might be altered by radiotherapy, we measured cAMP level and PKA activity in DRG and the spinal cord following radiotherapy treatment. The results showed that radiotherapy treatment significantly reduced TCI-induced increase of cAMP concentration as well as PKA activity in DRG and the spinal cord (Figure 4).

### 3.5. Radiotherapy Reduced TCI-Induced Activity of IL-1*β* and TNF-*α* in the Spinal Cord

**Figure 5:**
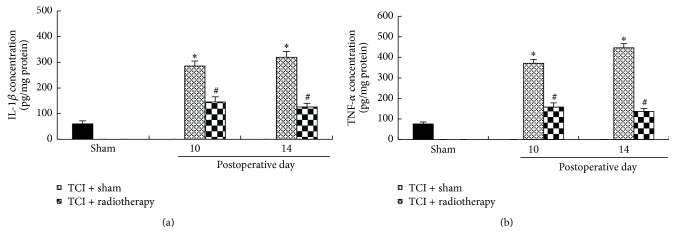
Radiotherapy reduced TCI-induced increase of IL-1*β* and TNF-*α* in the spinal cord. IL-1*β* and TNF-*α* concentrations were analyzed by ELISA. The tissues were collected on postoperative days 10 and 14, that is, 1 and 5 days after radiotherapy, respectively. Four samples were included in each group and each sample included a segment of the spinal cord at L_4_-L_5_. ^*∗*^ *P* < 0.05 versus sham; ^#^ *P* < 0.05 versus TCI + sham.

We have recently shown that IL-1*β* and TNF-*α* levels in the spinal cord were significantly increased in a time-dependent manner after TCI treatment [25]. To test whether radiotherapy could affect TCI-induced increase of IL-1*β* and TNF-*α*, we measured levels of IL-1*β* and TNF-*α* in the spinal cord after radiotherapy on postoperative days 10 and 14 (1 and 5 days after radiotherapy). Our results showed that radiotherapy treatment significantly reduced TCI-induced increase of IL-1*β* and TNF-*α* in the spinal cord (Figure 5).

## 4. Discussion

This study demonstrates that radiotherapy is an effective treatment approach for treating bone cancer pain. The cAMP-PKA signaling pathway may be a mechanism that underlies the analgesic effect of radiotherapy in bone cancer pain. Radiotherapy suppresses TCI-induced painful behaviors, thermal hyperalgesia, and mechanical allodynia and alleviates TCI-induced massive bone destruction. Radiotherapy reduces TCI-induced increased expression of PKA mRNAs in DRG as well as the increased level of cAMP concentration and PKA activity in both DRG and the spinal cord. In addition, radiotherapy results in a significant decrease of IL-1*β* and TNF-*α* activity in the spinal cord. These findings suggest that radiotherapy treatment may suppress TCI-induced hyperalgesia and allodynia by inhibiting the cAMP-PKA signaling pathway in DRG and the spinal cord.

Pain is one of the most prominent symptoms in clinically advanced cancer patients. Approximately 50% of these patients experience moderate to severe pain [1]. Mechanisms of cancer pain are thought to be complex and may involve a combination of inflammation, nerve injury, and other unique factors [26]. Considerable evidence of cancer pain treatment shows that radiotherapy effectively relieves pain in up to 95% of patients and can maintain the level of analgesia in more than 70% of the patients for up to three months [27–29]. Previous studies have shown that activation of the cAMP-PKA signaling pathway contributes to noxious stimulus-induced peripheral and central sensitization [30–32]. We have found that* in vivo* chronic compression of DRG or* in vitro* acute DRG dissociation resulted in activation of the cAMP-PKA signaling pathway. Continued activation of the cAMP-PKA signaling pathway is required to maintain hyperexcitability of the DRG neurons and behaviorally expressed hyperalgesia in these two different injury-related stress conditions [15, 17]. Recently, we have confirmed that activation of the cAMP-PKA pathway plays an essential role in the induction and maintenance of bone cancer pain [19, 25]. Here, we provide direct evidence that the elevated activity of cAMP-PKA signaling pathway is significantly decreased by radiotherapy in a bone cancer pain model. These results support an idea that radiotherapy may suppress bone cancer pain through inhibition of abnormal activation of cAMP-PKA signaling pathway, suggesting a new mechanism for the radiotherapy of bone cancer pain.

The proinflammatory cytokines are activators of the cAMP-PKA pathway in primary afferent neurons [25, 33–35]. During tumor growth and development of bone cancer pain, certain proinflammatory cytokines such as TNF-*α* and IL-1*β* are activated and released from the astrocytes and microglial cells and contribute to bone cancer pain [25, 36, 37]. Radiotherapy can reduce these proinflammatory cytokines and inhibit activation of the cAMP-PKA signaling pathway and thus result in relief of bone cancer pain. These findings suggest that the cytokines are also targets that may be responsible for radiotherapy-induced analgesia. In addition, our results showed that radiotherapy resulted in less bone loss in TCI rats. This is consistent with the previous finding that radiotherapy with high dose may kill part of the tumor cells or reduce their activation and thus delay the destruction of the bone structure [38, 39]. These findings indicate that radiotherapy may reduce both pain and loss of bone structure following TCI treatment.

## 5. Conclusions

Our results demonstrate that radiotherapy can effectively suppress bone cancer pain probably through inhibition of activation of cAMP-PKA signaling pathway in the primary sensory neurons and the spinal cord. This study may suggest a new mechanism underlying radiotherapy-induced analgesia of bone cancer pain and support the clinical use of radiotherapy in treatment of certain cancer pain conditions.

## Figures and Tables

**Table 1:** Primer sequences of the genes studies for RT-PCR.

Primer name	Forward	Reverse
PKA-RII	5-ACCTCAGACGGCTCCCTTTG-3	5-CGTCTCCAACCGCATAAGCAG-3
PKA-C	5-ACCTTGGGAACGGGTTCCTTCG-3	5-TACACCCAATGCCCACCAGTCC-3
*β*-actin	5-TCTACAATGAGCTGCGTGTG-3	5-AATGTCACGCACGATTTCCC-3
